# Engineering and application of synthetic *nar* promoter for fine-tuning the expression of metabolic pathway genes in *Escherichia coli*

**DOI:** 10.1186/s13068-018-1104-1

**Published:** 2018-04-07

**Authors:** Hee Jin Hwang, Sang Yup Lee, Pyung Cheon Lee

**Affiliations:** 1 Department of Molecular Science and Technology, Ajou University, Woncheon-dong, Yeongtong-gu, Suwon, 16944 South Korea; 2 Department of Chemical and Biomolecular Engineering, KAIST, Daejeon, 34141 South Korea

**Keywords:** *nar* promoter, Oxygen-dependent promoter, Lactate, 2,3-Butanediol, Promoter engineering

## Abstract

**Background:**

Promoters regulate the expression of metabolic pathway genes to control the flux of metabolism. Therefore, fine-tuning of metabolic pathway gene expression requires an applicable promoter system. In this study, a dissolved oxygen-dependent *nar* promoter was engineered for fine-tuning the expression levels of biosynthetic pathway enzymes in *Escherichia coli*. To demonstrate the feasibility of using the synthetic *nar* promoters in production of biochemicals in *E. coli*, the d-lactate pathway consisting of one enzyme and the 2,3-butanediol (BDO) pathway consisting of three enzymes were investigated.

**Results:**

The spacer sequence of 15 bp between the − 35 and − 10 elements of the upstream region of the wild-type *nar* promoter was randomized, fused to the GFP gene, transduced into *E. coli*, and screened by flow cytometry. The sorted synthetic *nar* promoters were divided into three groups according to fluorescence intensity levels: strong, intermediate, and weak. The selected three representative *nar* promoters of strong, intermediate, and weak intensities were used to control the expression level of the d-lactate and 2,3-BDO biosynthetic pathway enzymes in *E. coli*. When the *ldhD* gene encoding d-lactate dehydrogenase was expressed under the control of the strong synthetic *nar* promoter in fed-batch cultures of *E. coli*, the d-lactate titers were 105.6 g/L, 34% higher than those using the wild-type promoter (79.0 g/L). When the three 2,3-BDO pathway genes (*ilvBN*, *aldB*, and *bdh1*) were expressed under the control of combinational synthetic *nar* promoters (strong–weak–strong) in fed-batch cultures of *E. coli*, the titers of 2,3-BDO were 88.0 g/L, 72% higher than those using the wild-type promoter (51.1 g/L).

**Conclusions:**

The synthetic *nar* promoters, which were engineered to have strong, intermediate, and weak intensities, were successfully applied to metabolic engineering of d-lactate and 2,3-BDO pathways in *E. coli*. By controlling expression levels of d-lactate and 2,3-BDO pathway enzymes using the synthetic *nar* promoters, the production of d-lactate and 2,3-BDO was increased over that using the wild-type promoter by 34 and 72%, respectively. Thus, this synthetic promoter module system will support the improved production of biochemicals and biofuels through fine-tuning of gene expression levels.

## Background

Synthetic biology aims to develop desired biological system through the rational design of synthetic parts/modules, including promoters, RNAs, and scaffolds [1–4]. Gene expression can be controlled utilizing several factors, such as promoters, transcription factors, and plasmid copy numbers [5, 6]. Among them, promoter engineering has been proposed as one of the most efficient ways of fine-tuning transcriptional control in *Escherichia coli*, *Corynebacterium glutamicum*, *Bacillus subtilis*, and yeasts [3–10]. For example, the *E. coli* strain with engineered l-phenylalanine-responsive promoter could produce fourfold higher titer of phenylalanine than wild-type promoter [11], and the engineered *tac* promoter library could decrease leakage of antibody fragment expression in *E. coli* [12].

Recently, a dissolved oxygen (DO)-dependent *nar* promoter was successfully applied to express the d-lactate, 2,3-butanediol (BDO), and 1,3-propanediol (PDO) pathway enzymes in *E. coli* [13]. However, when a multienzyme biosynthetic pathway was reconstructed in heterologous host cells, individual expression of each pathway enzyme needed to be finely controlled; assembly or organization of multienzyme systems could significantly influence metabolic channeling, and thus suboptimal assembly or organization would cause accumulation of unwanted metabolic intermediates in multi-step enzyme reactions [14, 15]. Even single-enzyme metabolic pathways also should be considered for fine-tuning of expression, because expression level frequently affects end-product formation due to inclusion body formation [16].

**Fig. 1:**
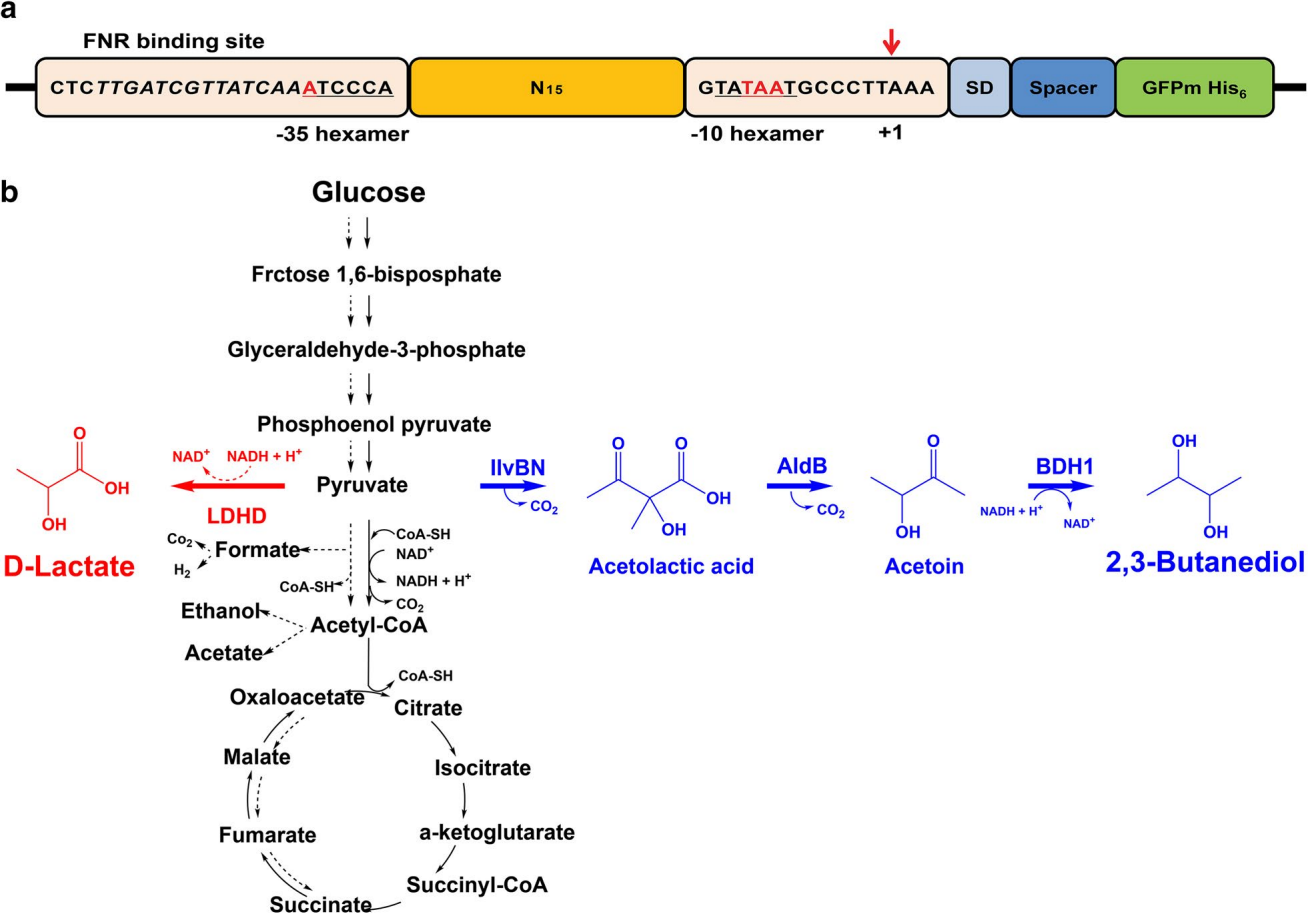
**a** Schematic diagram for construction of the synthetic *nar* promoter library. The 15-bp spacer sequence (indicated by consecutive 15 Ns) between the − 35 and − 10 elements of the wild-type *nar* promoter was randomized. An FNR binding site is shown in italics; the − 35 and − 10 elements are indicated by underlines; a red arrow indicates a transcription start site (+ 1); SD, Shine–Dalgarno sequence, GFPm; modified green fluorescence protein with His_6_ tag. **b** Reconstructed d-lactate and 2,3-butanediol (2,3-BDO) biosynthesis pathways in *E. coli*. The d-lactate pathway (red) consists of *ldhD* gene encoding d-lactate dehydrogenase (LDHD); the 2,3-BDO pathway (blue) consists of *ilvBN* gene encoding acetohydroxybutanoate synthase/acetolactate synthase (IlvBN), *aldB* gene encoding acetolactate decarboxylase (AldB), and *bdh1* encoding butanediol dehydrogenase (BDH1)

Compared to other commonly used strong promoters such as *lac* or *araBAD*, the intensity of the wild-type *nar* promoter is relatively weak [13]; therefore, engineering of the wild-type *nar* promoter was required for fine control of target pathway gene expression. In this study, in order to generate synthetic *nar* promoters of diverse strengths, a synthetic *nar* promoter library was constructed by randomization of the spacer region sequence (15 bp) located between the consensus sequence − 35 and − 10 elements of the wild-type *nar* promoter (Fig. 1a). Characterization of the selected three synthetic promoters showing weak, intermediate, and strong intensities was performed through transcriptional expression level and GFP fluorescence intensity assays. Then, the selected three synthetic promoters were applied to the expression of the d-lactate pathway consisting of one gene and the 2,3-BDO biosynthesis pathway consisting of three genes (Fig. 1b). We demonstrated that the production of d-lactate and 2,3-BDO was improved by tuning the expression of their pathway genes under three different strengths of the synthetic *nar* promoters in *E. coli*.

## Results

### Construction, screening, and strength analysis of a synthetic *nar* promoter library of diverse strengths

The 15-bp spacer region sequence between the − 35 and − 10 elements of the wild-type *nar* promoter was randomized using degenerated primers to construct a synthetic *nar* promoter library of diverse strengths. The randomized synthetic promoters were fused by PCR to a DNA fragment consisting of a Shine–Dalgarno sequence, spacer, and His_6_-tagged GFPm as a reporter protein for screening based on the fluorescence intensity of the expressed GFPm (Fig. 1a). The randomized promoter fragments were then ligated with a pSTVM plasmid (Table 1) and then transduced into *E. coli* TOP10 cells.

**Table 1:** Bacterial strains and plasmids used in this study

Strains and plasmids	Relevant properties	Source or reference
Strains		
*E. coli* TOP10	F-*mcrAΔ(mrr*-*hsdRMS*-*mcrBC) φ80lacZΔM15 ΔlacX74 nupG recA1 araD139 Δ(ara*-*leu)7697 galE15 galK16 rpsL(Str*^*R*^*)* endA1	Invitrogen
W023	W *ΔldhA ΔpflB ΔadhE ΔlpdA*::*K.p. lpdE354* *K Δmdh ΔarcA gltAR164L*	[13, 21]
*Lactobacillus citreum*	Source for *ldhD*	KCTC3721
*Lactococcus lactis* subsp. *lactis*	Source for *aldB*	KCTC3899
*Saccharomyces cerevisiae* S288c	Source for *bdh1*	ATCC 204508
Plasmids		
pUCM	Cloning vector modified from pUC19; constitutive *lac* promoter, Ap^R^	[23]
pUCM-*gfpm*	Constitutive expressed *gfpm* gene with *lac* promoter	This study
pUCN	Cloning/expression vector having inducible wild-type *nar* promoter, Amp^R^	[13]
pUCN-*gfpm*	Inducible expressed *gfpm* gene with *nar* promoter	This study
pQE-*gfpm*	Inducible expressed *gfpm* gene with T5 promoter	[22]
pUCNr	Cloning/expression vector having *rop* gene and wild-type *nar* promoter, Amp^R^	This study
pUCNrS	Cloning/expression vector having *rop* gene and strong *nar* promoter (S3-2-64), Amp^R^	This study
pUCNrI	Cloning/expression vector having *rop* gene and intermediate *nar* promoter (W2U-30), Amp^R^	This study
pUCNrW	Cloning/expression vector having *rop* gene and weak *nar* promoter (W2L-29), Amp^R^	This study
NrSL	Inducible expressed *ldhD* gene from *L. citreum* on pUCNrS	This study
NrIL	Inducible expressed *ldhD* gene from *L. citreum* on pUCNrI	This study
NrWL	Inducible expressed *ldhD* gene from *L. citreum* on pUCNrW	This study
NrSi	Inducible expressed *ilvBN* gene from *E. coli* on pUCNrS	This study
NrIi	Inducible expressed *ilvBN* gene from *E. coli* on pUCNrI	This study
NrWi	Inducible expressed *ilvBN* gene from *E. coli* on pUCNrW	This study
NrSa	Inducible expressed *aldB* gene from *L. lactis* with strong *nar* promoter	This study
NrIa	Inducible expressed *aldB* gene from *L. lactis* with intermediate *nar* promoter	This study
NrWa	Inducible expressed *aldB* gene from *L. lactis* with weak *nar* promoter	This study
NrSb	Inducible expressed *bdh1* gene from *S. cerevisiae* with strong *nar* promoter	This study
NrIb	Inducible expressed *bdh1* gene from *S. cerevisiae* with intermediate *nar* promoter	This study
NrWb	Inducible expressed *bdh1* gene from *S. cerevisiae* with weak *nar* promoter	This study
pSTVM2	Cloning/expression vector removing *lac* promoter, Cm^R^	[13]
pSTVM2- SNPL-gfpm	*gfpm* expression vector with synthetic *nar* promoter library	This study
pSTVM-*gfpm*	Constitutive expressed gfpm gene with constitutive *lac* promoter on pSTVM2	This study
Ni	Inducible expressed *ilvBN* gene with wild-type *nar* promoter on pSTVM2	[13]
Si	Inducible expressed *ilvBN* gene with strong *nar* promoter on pSTVM2	This study
Ii	Inducible expressed *ilvBN* gene with intermediate *nar* promoter on pSTVM2	This study
Wi	Inducible expressed *ilvBN* gene with weak *nar* promoter on pSTVM2	This study
NiNa	Individually inducible expressed *ilvBN and aldB* genes with wild-type *nar* promoter on pSTVM2	[13]
SiSa	Individually inducible expressed *ilvBN* and *aldB* genes with strong *nar* promoter on pSTVM2	This study
IiIa	Individually inducible expressed *ilvBN* and *aldB* genes with intermediate *nar* promoter on pSTVM2	This study
SiWa	Individually inducible expressed *ilvBN* with strong promoter and *aldB* with weak *nar* promoter on pSTVM2	This study
NNN	Inducible expressed *ilvBN, aldB,* and *bdh1* genes under each *nar* promoter on pSTVM2	[13]
SWS	Individually inducible expressed *ilvBN* with strong promoter, *aldB* with weak promoter, and *bdh1* with strong promoter	This study

**Fig. 2:**
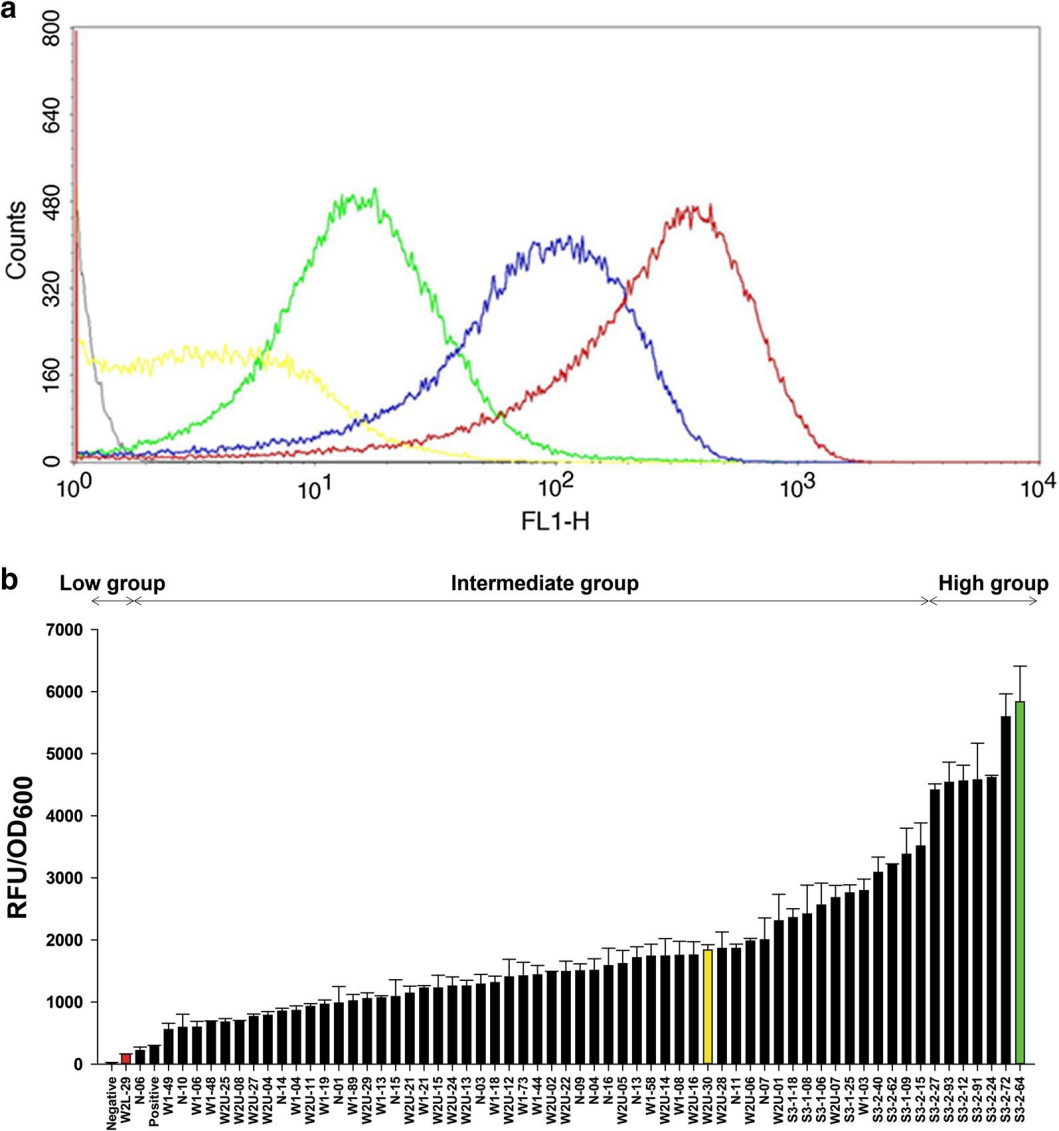
**a** FACS analysis of *E. coli* cells containing the synthetic *nar* promoter library. Histograms of sorted cell libraries and control cells are represented by colored lines. Black: negative control (empty vector), yellow: sorted cells showing weak intensities, green: positive control (a wild-type *nar* promoter), blue: sorted cells showing intermediate intensities, and red: sorted cells showing strong intensities. **b** Promoter strength analysis of the synthetic *nar* promoters. GFP fluorescence intensities of the collected 68 clones having synthetic *nar* promoters were measured by spectrofluorometer and normalized as relative fluorescence unit (RFU)/OD_600_. Representative strong (S3-2-64), intermediate (W2U-30), and weak (W2L-29) *nar* promoters are represented by green, yellow, and red bars, respectively

The library (4.59 × 10^10^ cell size), showing diverse fluorescence intensities, was sorted into three groups (low, intermediate, and high strength) by FACS in three rounds of sorting. The 1st round sorting was carried out with two collections of the upper and lower 1% of fluorescence intensity signals. The collected cells were grown on LB+cm agar plates and submitted to 2nd round sorting. After repeating this for 3rd round sorting, clones showing high (a red line in Fig. 2a), immediate (a blue line), and low (a yellow line) fluorescence intensities were collected and further analyzed.

After reconfirmation of the GFP fluorescence intensities of 300 randomly selected clones from the 3 groups (100 clones from each group), fluorescences of 68 distinguishable clones were measured and compared based on relative fluorescence units (RFU)/OD_600_ (Fig. 2b). Based on the normalized values of RFU/OD_600_, synthetic *nar* promoters were grouped into strong (> 5000 RFU/OD_600_), intermediate (1000–2000 RFU/OD_600_), and weak (< 1000 RFU/OD_600_) groups. From each group, a representative synthetic *nar* promoter of strong (S3-2-64), intermediate (W2U-30), and weak (W2L-29) fluorescence was chosen for further analysis. The strength of the strong synthetic *nar* promoter (S3-2-64) and the intermediate *nar* promoter (W2U-30) were 19.8 and 6.2 times higher than that of the wild-type *nar* promoter, respectively, while the strength of the weak *nar* promoter (W2L-29) was 1.8 times weaker than that of the wild-type *nar* promoter based on values of RFU/OD_600_.

### Characterization of the three representative synthetic *nar* promoters

**Fig. 3:**
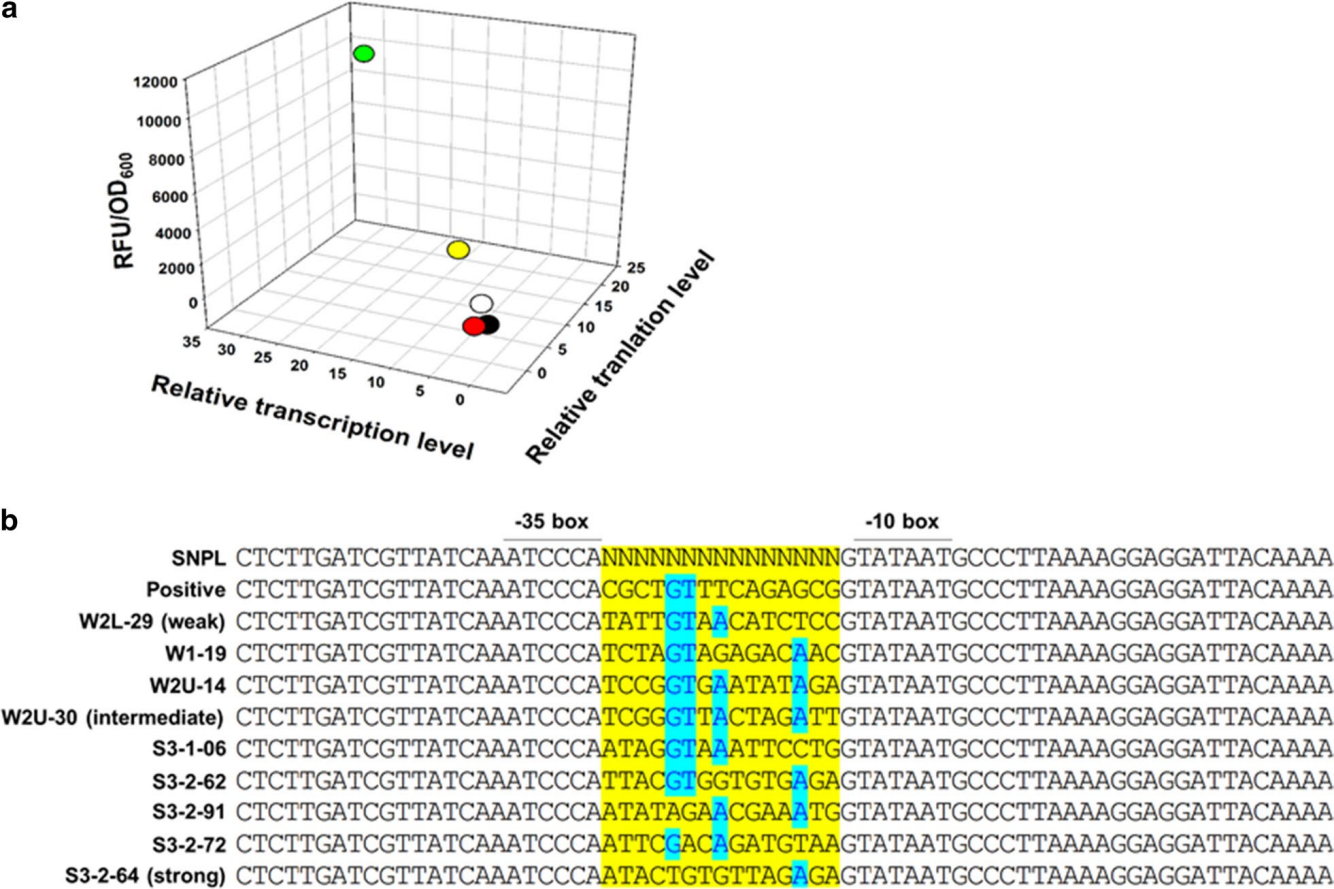
**a** Evaluation of synthetic promoter strength. Correlation among relative mRNA expression levels (*x*-axis), relative protein expression levels (*y*-axis), and GFP fluorescence intensities (*z*-axis). Green circle, a strong *nar* promoter; yellow circle, an intermediate *nar* promoter; red circle, a weak *nar* promoter, white circle; a constitutive *lac* promoter, black circle; a wild-type *nar* promoter. **b** Sequence analysis of the representative synthetic *nar* promoters. The yellow box indicates the randomly mutated spacer sequence between the − 35 box and − 10 box. The blue boxes indicate consensus bases in the spacer region of *nar* promoters

The three representative *nar* synthetic promoters were then characterized in detail by analyzing levels of transcription, protein expression, and fluorescence of GFPm (Fig. 3a). In transcriptional analysis, qRT-PCR was carried out with a *cysG* gene encoding siroheme synthase [17] as a reference in order to evaluate ΔΔ*C*_t_ values of GFPm expression under the control of the wild-type, strong, intermediate, and weak *nar* promoters. The ΔΔ*C*_t_ values of the strong, intermediate, and weak *nar* promoters were 29.4 ± 5.6, 8.4 ± 1.0, and 2.3 ± 0.4, respectively. Unexpectedly, the ΔΔ*C*_t_ value of the weak *nar* promoter was positive, indicating a higher transcription level than that of the wild-type *nar* promoter. Next, protein expression levels were examined by western blotting with endogenous GAPDH as a reference. The protein expression levels of GFPm under the control of the strong, intermediate, and weak *nar* promoters were approximately 19.7, 7.11, and 0.37 times higher than that of the wild-type *nar* promoter. Notably, the protein expression levels of the strong and intermediate *nar* promoters were 4.6 and 1.7 times higher, respectively, than that of the constitutive *lac* promoter. Finally, the fluorescence intensities of GFPm under the control of the strong, intermediate, and weak *nar* promoters were 10,017 ± 915, 2305 ± 360, and 218 ± 18 RFU/OD_600_, respectively.

The randomly mutated spacer regions of the nine *nar* promoters including three representatives were sequenced and compared. One distinguishable difference between the synthetic and wild-type *nar* promoters was a GC content in the space sequences: lower GC contents were observed in all synthetic promoters (strong: 33.3%; intermediate: 40%; weak: 33.3%) than in the wild-type *nar* promoter (60%) (Fig. 3b). A conserved sequence (GTN[A/G]N) located between the − 24 and − 20 positions was observed in seven clones representing intermediate and weak promoters, but not in that of the strong promoter. It has been shown that strong promoters tend to have AT-rich spacers, which help flexibility and bendability of DNA structures [18]. This is true for the strong *nar* promoter because it has AT-rich spacers and lacks the conserved sequence present in the weaker promoters.

### Comparison of d-lactate production with synthetic *nar* promoters of different strengths

**Fig. 4:**
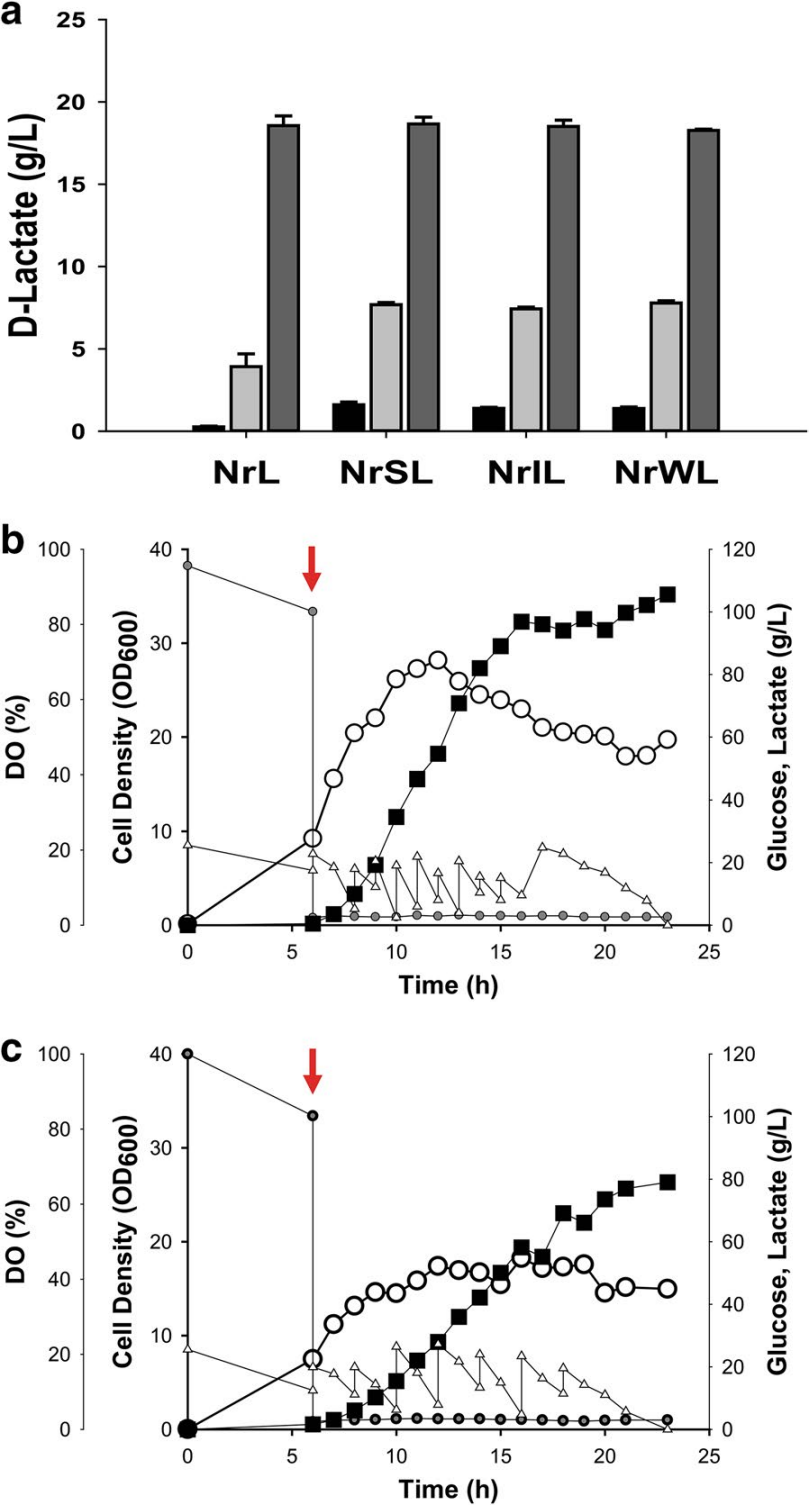
**a** Comparison of d-lactate production by each promoter in flask-scale fermentations. The black, light gray, and dark gray indicate production of d-lactate after 4, 8, and 20 h, respectively. The error bars show the standard deviations from triplicate experiments. Production of d-lactate under control of strong synthetic *nar* promoter (**b**) and the wild-type *nar* promoter (**c**) in fed-batch fermentations. Black square, d-lactate; hollow circle, cell growth; hollow triangle, glucose; solid gray circle, DO level; red arrows, the time of DO downshift (induction)

In order to investigate the effect of *nar* promoter strength on metabolic pathway flux in *E. coli*, a d-lactate pathway consisting of one d-lactate dehydrogenase was first chosen. The *ldhD* gene encoding d-lactate dehydrogenase from *L. citreum* was cloned into pUCNrS, pUCNrI, pUCNrW, and pUCN (Table 1), to be expressed under the control of the four *nar* promoters: strong, intermediate, weak, and wild-type. The four *ldhD* gene-expression plasmids (NrSL, strong; NrIL, intermediate; NrWL, weak; NrL, wild-type) were transduced into *E. coli*, and then the four recombinant *E. coli* strains were microaerobically grown in flasks containing 20 g/L glucose as a carbon source [13]. After a 20-h cultivation, d-lactate titers were measured to be 18.6 ± 0.6 in *E. coli* having NrL, 18.7 ± 0.4 in NrSL, 18.5 ± 0.4 in NrIL, and 18.3 ± 0.1 in NrWL (Fig. 4a). Because d-lactate titers were similar in all four *E. coli* strains, two *E. coli* strains having NrL and NrSL were chosen and fed-batch fermentation with glucose as a carbon source was carried out to investigate the effect of the strength of *nar* promoters (strong vs. wild-type) on d-lactate production. When the DO-controlled fed-batch fermentation of *E. coli* strain having NrSL was carried out as described in our previous study [13], 105.6 g/L of d-lactate was obtained after a 23-h cultivation. The d-lactate yield and productivity were 0.71 g/g-glucose and 4.59 g/L/h, respectively (Fig. 4b). In comparison, the *E. coli* strain with NrL produced 79.0 g/L of d-lactate with d-lactate yield of 0.67 g/g-glucose and productivity of 3.47 g/L/h (Fig. 4c). This result supports that higher expression of d-lactate dehydrogenase under the control of the synthetic strong *nar* promoter directed more metabolic flux into d-lactate biosynthesis in *E. coli*. Consequently, controlling d-lactate dehydrogenase with the strong *nar* promoter enhanced d-lactate titers by 34% compared to those of the wild-type *nar* promoter (105.6 g/L vs. 79.0 g/L).

### Comparison of acetoin and 2,3-BDO production with the synthetic *nar* promoters of different strengths

**Fig. 5:**
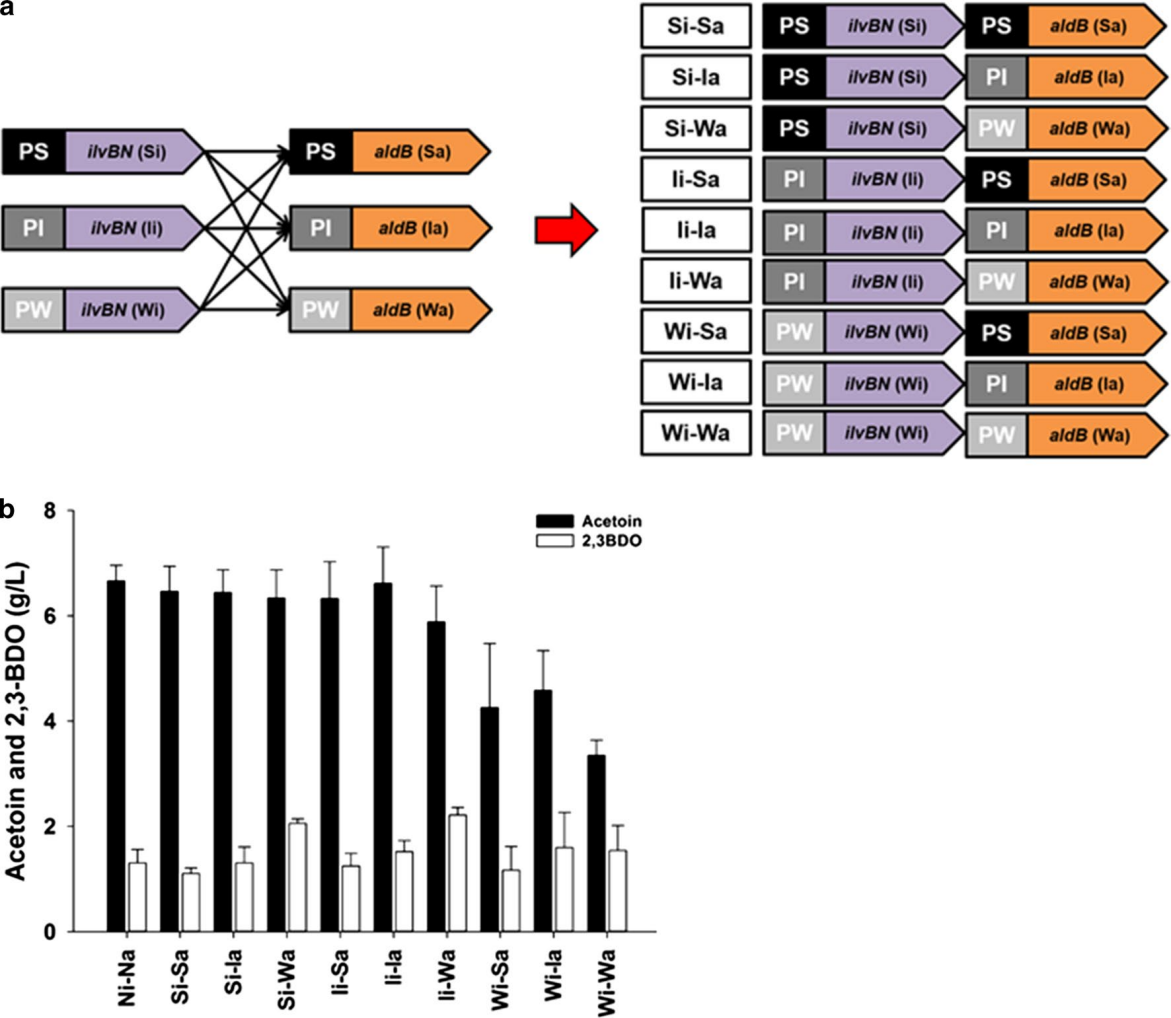
Comparison of acetoin production with the expression of *ilvBN* and *aldB* under control of the 3 synthetic *nar* promoters in flask cultures. **a** Combination of expression modules of *ilvBN* and *aldB* genes. Modules are Si, Ii, Wi, Sa, Ia, and Wa; S stands for strong promoter, I for intermediate promoter, W for weak promoter, i for *ilvBN*, and a for *aldB*. **b** Titer of acetoin obtained by expressing 10 modules in flask cultures. The positive control Ni–Na is a module expressing the *ilvBN* and *aldB* genes with a wild-type *nar* promoter. Black and white bars indicate acetoin and 2,3-BDO, respectively

As a second demonstration of the feasibility of using the synthetic *nar* promoters in production of biochemicals in *E. coli*, acetoin, consisting of two enzymes, and the 2,3-butanediol (2,3-BDO) pathway, consisting of three enzymes, were investigated using the three representative *nar* promoters. First, the production of acetoin, a 2,3-BDO pathway intermediate (Fig. 1b), was investigated. In order to reconstruct the heterologous acetoin pathway in *E. coli*, two acetoin pathway genes, *ilvBN* from *E. coli* and *aldB* from *L. lactis*, were modified to be expressed under the control of the three synthetic *nar* promoters by cloning each gene into pUCNrS, pUCNrI, and pUCNrW. Next, the resulting 6 expression modules (Si, Ii, Wi, Sa, Ia, and Wa, where S stands for strong promoter, I for intermediate promoter, W for weak promoter, i for *ilvBN,* and a for *aldB*) were combined to produce 9 expression vectors (Fig. 5a).

When 10 expression modules including a positive control module Ni–Na (*ilvBN* and *aldB* expressed with a wild-type *nar* promoter) were expressed in flask cultures of *E. coli*, the expression of the *ilvBN* gene with strong and intermediate synthetic *nar* promoters (Si and Ii) produced an average of 6.30 g/L of acetoin regardless of promoter strength for expressing the *aldB* gene. However, the expression of *ilvBN* with the weak *nar* promoter (Wi) produced the lowest acetoin production (3.4 ± 0.3 g/L) when the *aldB* gene was coexpressed with the weak *nar* promoter (Wa) (Fig. 5b). Unexpectedly, when a heterologous acetoin pathway was reconstructed in *E. coli*, the end-product acetoin was to a limited degree transformed into 2,3-BDO by unknown factors [19]. Therefore, when the total summed amount of acetoin and 2,3-BDO produced by combination of the 10 modules was taken into consideration, Si–Wa and Ii–Ia combinations exhibited better production than the others (8.4 ± 0.5 and 8.1 ± 0.6 g/L, respectively).

**Fig. 6:**
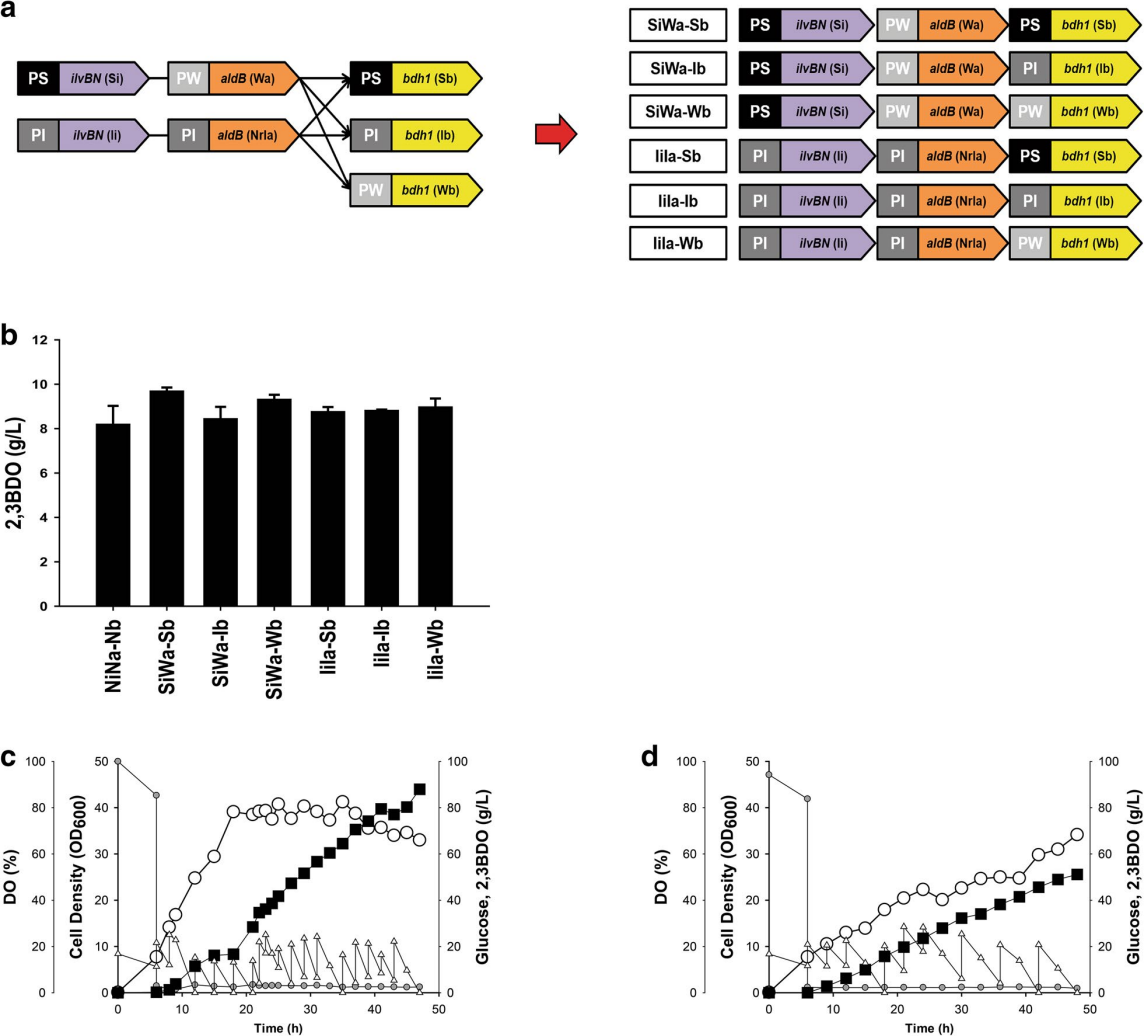
Comparison of 2,3-BDO production with the expression of *ilvBN, aldB*, and *bdh1* under control of the 3 synthetic *nar* promoters. **a** Combination of expression modules of *ilvBN, aldB*, and *bdh1* genes. Modules are Si, Ii, Wi, Sa, Ia, Wa, Sb, Ib, and Wb; S stands for strong promoter, I for intermediate promoter, W for weak promoter, i for *ilvBN*, a for *aldB*, and b for *bdh1*. **b** Titer of 2,3-BDO obtained by expressing 7 modules in flask cultures. The positive control NiNa-Nb is a module expressing *ilvBN, aldB*, and *bdh1* genes with a wild-type *nar* promoter. Fed-batch cultivation of *E. coli* expressing SWS (**c**) and NNN (**d**) modules. The SWS module expressed *ilvBN* with a strong, *aldB* with a weak, and *bdh1* with a strong *nar* promoter. The positive control NNN module expressed *ilvBN, aldB*, and *bdh1* genes with a wild-type *nar* promoter. Black square, 2,3-BDO; hollow circle, cell growth; hollow triangle, glucose; solid gray circle, DO level; red arrows, the time of DO downshift (induction)

Next, the two selected acetoin-producing combination modules, Si–Wa and Ii–Ia, were assembled as independent expression modules into one plasmid pSTVM2, resulting in SiWa and IiIa (Table 1). In order to reconstruct a 2,3-BDO pathway on the two-plasmid system, SiWa and IiIa were coexpressed with *bdh1* from *S. cerevisiae* under the control of the 3 synthetic *nar* promoters (6 expression combinations: Si, Ii, Wi, Sa, Ia, Wa, Sb, Ib, and Wb, where S stands for strong, I for intermediate, W for weak, i for *ilvBN*, a for *aldB*, and b for *bdh1*; Fig. 6a) in flask cultures. Among seven complementations including a wild-type *nar* promoter module (NiNa–Nb), the SiWa–Sb combination module produced the highest titer of 2,3-BDO (9.7 ± 0.2 g/L), followed by the SiWa–Wb module (Fig. 6b). This best combination modules along with the wild-type *nar* promoter module as a positive control were then assembled as an independent expression module into one plasmid pSTVM2, resulting in SWS and NNN. After fed-batch cultivation of *E. coli* expressing NNN and SWS, the SWS module produced 88.0 g/L of 2,3-BDO (Fig. 6c), while the NNN module produced 51.1 g/L of 2,3-BDO (Fig. 6d). This fine-tuning of each 2,3-BDO pathway enzyme expression enhanced the 2,3-BDO titer by 72%. Even though conversion yields (g/g-glucose) were similar [0.33 (NNN) vs. 0.35 (SWS)], volumetric productivity of 2,3-BDO obtained by expressing SWS was 1.87 g/L/h, which was 75% higher than the 1.07 g/L/h obtained by expressing NNN.

## Discussion

A promoter is one cellular strategy for controlling the flux of metabolism by regulating the expression of metabolic pathway genes. Therefore, fine-tuning of metabolic pathway gene expression requires an applicable promoter system. In our previous study, a DO-dependent *nar* promoter was successfully applied to control expressions of biochemical biosynthetic pathway enzymes in *E. coli* [13]. However, more fine-tuning of expressions of biochemical biosynthetic pathway enzymes is necessary to enhance yield and titer of a target biochemical or biofuel by metabolic flux channeling. Therefore, in this study, 3 synthetic *nar* promoters showing weak (W2L-29), intermediate (W2U-30), and strong (S3-2-64) intensities were constructed by randomization of the spacer region sequence (15 bp) located between the consensus sequence − 35 and − 10 elements of the wild-type *nar* promoter (Fig. 1a). Analyses of transcription by qRT-PCR, protein expression by western blotting, and fluorescence by a GFPm reporter protein assay were in a good correlation with the apparent strengths of the 3 synthetic *nar* promoters (Fig. 3a). Sequence analysis showed that these synthetic *nar* promoters have relatively higher AT contents than the wild-type *nar* promoter and higher variations at the − 24, − 20, and − 14 sequence sites (Fig. 3b). The observed features of the synthetic *nar* promoters are well supported by other research reporting that the AT-rich sequences of the spacer region affected the strength of the promoter through structural changes [18, 20].

To evaluate the general use of synthetic promoters in biochemical production, the 3 synthetic *nar* promoters were used to express d-lactate and 2,3-BDO pathway enzymes. Among the 3 synthetic *nar* promoters, the expression of the *ldhD* gene under the control of the strong synthetic *nar* promoter on a low-copy plasmid produced the highest amount (105.6 g/L) of d-lactate by fed-batch fermentation. This titer is slight lower than the 113.1 g/L of d-lactate which was obtained by fed-batch fermentation [13] by expression of the *ldhD* gene with the wild-type *nar* promoter on a high-copy plasmid. This suggests that the *ldhD* expression level may be highly correlated with titer of d-lactate. The correlation of expression level and production titer was also high in the production of 2,3-BDO. In a similar manner to d-lactate pathway gene expression, each of the three 2,3-BDO biosynthesis pathway genes (*ilvBN*, *aldB*, and *bdh1*) were modified to be under the control of the synthetic *nar* promoters of different strengths and combinatorially expressed. In the case of the acetoin pathway, which is a precursor of 2,3-BDO, controlling *ilvBN* gene expression was critical in biosynthesis flux because the expression of *ilvBN* with a weak *nar* promoter resulted in lower titers of acetoin than expression with strong or intermediate promoters regardless of the promoter strength for expressing the *aldB* gene. The acetoin pathway was further extended to 2,3-BDO by controlling expression of the *bdh1* gene with the 3 synthetic *nar* promoters. The best combinatorial expression module for 2,3-BDO was a strong promoter for *ilvBN* (Si), weak promoter for *aldB* (Wa), and strong promoter for *bdh1* (Sb). The expression module (Si + Wa + Sb) produced 88.0 g/L of 2,3-BDO in fed-batch fermentation, which was 72% higher than the 51.1 g/L obtained by expressing the 3 enzymes with a wild-type *nar* promoter.

## Conclusions

In this study, the synthetic *nar* promoters, which were engineered to have strong, intermediate, and weak intensities, were successfully applied to metabolic engineering of the d-lactate and 2,3-BDO pathways in *E. coli*. By controlling expression levels of one d-lactate enzyme and three 2,3-BDO pathway enzymes using the synthetic *nar* promoters, the production of d-lactate and 2,3-BDO was increased by 34 and 72%, respectively, compared with production using a wild-type *nar* promoter. This synthetic *nar* promoter module system will support the improved production of biochemicals and biofuels through the fine-tuning of gene expression levels in *E. coli*.

## Methods

### Strains

The *E. coli* TOP10 (Invitrogen, USA) strain was used for cloning and maintenance of plasmids. The *E. coli* W023 [13, 21] strain was used to produce d-lactate, acetoin, and 2,3-BDO in flask and fed-batch fermentation. The bacterial strains used in this study are listed in Table 1.

### Construction of the randomized *nar* promoter library

A *gfpm* gene encoding GFPm was amplified by PCR from pQE-*gfpm* [22] with gene-specific primers including ribosome binding site (RBS) and restriction enzyme sites, and then the PCR product was inserted between *Xma*I and *Not*I sites downstream of a wild-type *nar* promoter on pUCN [13] and pUCM plasmids [23]. In order to randomize the spacer sequence (15 bp) between the − 10 and − 35 elements of the *nar* promoter, two primers, *Xma*I-SNPL-gfpm-F and *Sph*I-gfpm-R (Table 2) were designed. The *Xma*I-SNPL-gfpm-F primer contains a restriction enzyme site (*Xma*I), the − 35 element, randomized 15-bp sequences (N_15_), the − 10 element, and the gfpm-specific sequence in order. The *Sph*I-gfpm-R primer contains a restriction enzyme site (*Sph*I) and the gfpm-specific sequence. The randomized *nar* promoter region and *gfpm* gene amplified by PCR were cloned into the *Xma*I and *Sph*I sites of the pSTVM2 plasmid [13], generating pSTVM2-SNPL-gfpm. The pSTVM2-SNPL-gfpm plasmids were transduced into *E. coli* TOP10 cells by electroporation and the transformants were incubated in 40 mL Luria–Bertani (LB) medium supplemented with 30 μg/L chloramphenicol [24] at 30 °C with shaking at 100 rpm.

**Table 2:** Primers used in this study

Primer name	Sequence (5′–3′)
For cloning	
*Xma*I-SNPL-gfpm-F	TCCC*CCCGGG*CTCTTGATCGTTATCAAATCCCANNNNNNNNNNNNNNNGTATAATGCCCTTAAAAGGAGGATTACAAAATGAGTAAAGGAGAAGAACT
*Sph*I-gfpm-R	ACAT*GCATGC*TTAGTGGTGGTGGTGGTGGTGTTTGTAGAGCTCATCGATGC
*Xma*I-gfpm-F	TCCC*CCCGGG*AGGAGGATTACAAAATGAGTAAAGGAGAAGAACTTTT
*Not*I-gfpm-R	TAAGAAT*GCGGCCGC*TTAGTGGTGGTGGTGGTGGTGTTTGTAGAGCTCATCGATGC
ldhD-citreum-F-XbaI	CTAG*TCTAGA*AGGAGGATTACAAAATGAAGATTTTTGCTTATGGT
ldhD-citreum-R-*Not*I	TTCCCTT*GCGGCCGC*TTAATACTTTACAGCAATACTT
ilvB-EC-F-XbaI	CTAG*TCTAGA*AGGAGGATTACAAAATGGCAAGTTCGGGCA
ilvN-EC-R-*Not*I	TTCCCTT*GCGGCCGC*TTACTGAAAAAACACCGCGAT
aldB-LL-F-XbaI	CTAG*TCTAGA*AGGAGGATTACAAAATGACAGAAATCACACAACTT
aldB-LL-R-*Not*I	TTCCCTT*GCGGCCGC*TCATTCAGCTACATCGATATC
bdh1-SC-F-*Xma*I	TCCC*CCCGGG*AGGAGGATTACAAAATGAGAGCTTTGGCATATTTC
bdh1-SC-R-*Not*I	TTCCCTT*GCGGCCGC*TTACTTCATTTCACCGTGATT
*Sph*I-pUC-F	ACAT*GCATGC*CCGACTGGAAAGCG
*Sph*I-pUC-R	ACAT*GCATGC*CGGTGTGAAATACCG
PstI-pUC-F	AAAA*CTGCAG*CCGACTGGAAAGCG
BamHI-pUC-R	CG*GGATCC*CGGTGTGAAATACCG
PnarS-R	TCTCTAACACAGTATTGGGATTTGATAACGATCAAG
PnarI-R	AATCTAGTAACCCGATGGGATTTGATAACGATCAAG
PnarW-R	GGAGATGTTACAATATGGGATTTGATAACGATCAAG
-10Pnar-F	GTATAATGCCCTTAAATCTAGA
pUCN-ori-fr	AGGAAGCGGAAGAGCG
pUCN-ori-r	GAAGATCCTTTGATCTTTTCTA
pET-ori-R	TTGAGATCCTTTTTTTCTGC
pET-rop-F	GGTGCGCATGATCGTG
pSTVM2-pUC-sub-USER-3-F	AGACAGUCATAAGTGCGG
pSTVM2-pUC-sub-USER-1-R	ATGCAACUCGTAGGACAG
pUC-sub-USER-1-F	AGTTGCAUCCCGACTGGAAAGCG
pUC-sub-USER-2-F	ATCCATGUCCCGACTGGAAAGCG
pUC-sub-USER-5-F	ATATGCGAUCCCGACTGGAAAGCG
pUC-sub-USER-2-R	ACATGGAUATGCGGTGTGAAATACC
pUC-sub-USER-5-R	ATCGCATAUATGCGGTGTGAAATACCG
pUC-sub-USER-3-R	ACTGTCUATGCGGTGTGAAATACCG
For qRT-PCR	
q-cysG-F	TTGTCGGCGGTGGTGATGTC
q-cysG-R	ATGCGGTGAACTGTGGAATAAACG
q-gfpm-F	AGAGGGTGAAGGTGATGCCA
q-gfpm-R	AGATGATCCGGATAACGCGC

Italic and underline letters represent a restriction enzyme site

### Screening of the randomized *nar* promoter library

*Escherichia coli* cells harboring pSTVM2-SNPL-gfpm were cultivated in 40 mL LB medium supplemented with 50 μg/mL Cm in a 100-mL flask at 30 °C with shaking at 100 rpm for 12 h. Cells were harvested by centrifugation at 7000 rpm for 10 min at 4 °C and washed twice with phosphate buffered saline (PBS, 137 mM NaCl, 2.7 mM KCl, 10 mM Na_2_HPO_4_, and 2 mM KH_2_PO_4_, pH 7.4). After washed cells were resuspended with PBS buffer, cells were subjected to a fluorescent activated cell sorter (FACS; MoFlo XDP, Beckman Coulter, FL). FACS-sorted cells were directly poured into fresh LB agar plates containing Cm (50 μg/mL) and incubated at 30 °C for 12 h. Cells scraped from the plates were then cultivated in 40 mL LB medium containing Cm (50 μg/mL) in a 100-mL flask and then subjected to the next round of FACS sorting following the same procedure mentioned above. After the third round of FACS sorting, colonies on LB agar plates were randomly selected to cultivate in 200 µL of LB+Cm medium in a 96-deep-well plate at 30 °C with shaking at 100 rpm overnight.

### Construction of plasmids

The nucleotide sequences of the primers used in construction of plasmids are listed in Table 2. Plasmid pUCN [13] was modified by adding the *rop* gene, which resulted in a low-copy number plasmid pUCNr with a wild-type *nar* promoter. Next, the wild-type *nar* promoter in pUCNr was replaced by one of the three representative synthetic *nar* promoters [S3-2-64 (strong), W2U-30 (moderate), W2L-29 (weak)] and amplified by PCR with each synthetic promoter-specific reverse primer and phosphorylated -10Pnar-F primer. The constructed plasmids were named pUCNrS (for S3-2-64), pUCNrI (for W2U-30), and pUCNrW (for W2L-29, weak promoter). The reporter *gfpm* gene was amplified by PCR from pQE-*gfpm* [22] with primers (*Xma*I-gfpm-F and *Not*I-gfpm-R) and cloned downstream of the *lac* promoter of pUCM, generating pUCM-gfpm. To obtain pSTVM-*gfpm*, the PCR-amplified *gfpm* gene containing the *lac* promoter was inserted between the *Bam*HI and *Eco*RI sites of the pSTVM2 plasmid [12]. The *ldhD*-encoding d-lactate dehydrogenase of *Leuconostoc citreum*, *ilvBN*-encoding acetohydroxy acid synthase of *E. coli*, *aldB*-encoding acetolactate decarboxylase of *Lactococcus lactis*, and *bdh1*-encoding butanediol dehydrogenase from *Saccharomyces cerevisiae* were amplified by PCR from the genomic DNAs of each strain, and then cloned downstream of the synthetic *nar* promoters of pUCNrS, pUCNrI, and pUCNrW (Table 2). For complementation experiments, the *ilvBN* gene was amplified by PCR with the synthetic *nar* promoter and a terminator, and then inserted between the *Pst*I and *Bam*HI sites of the pSTVM2 plasmid. To assemble two genes (*ilvBN* and *aldB*) encoding acetoin pathway enzymes and three genes (*ilvBN, aldB*, and *bdh1*) encoding 2,3-BDO pathway enzymes in pSTVM2, each gene was amplified by PCR with the synthetic *nar* promoter and a terminator, and then subcloned into pSTVM2 using the USER™ cloning method [25, 26].

### Flask and bioreactor fermentations

The recombinant *E. coli* strains harboring a plasmid or plasmids for production of d-lactate, acetoin, and 2,3-BDO were inoculated in 4 mL LB medium supplemented with 50 μg/mL Cm or/and 100 μg/mL ampicillin (Ap) at 37 °C overnight with shaking at 250 rpm. For flask cultivation, 100-mL flasks were filled with 40 mL LB medium containing 20 g/L glucose and appropriate antibiotics, and then were inoculated with 2% (v/v) seed culture. For d-lactate production, pH of the culture media was controlled by adding 10 g/L CaCO_3_. A *nar* promoter was induced by reducing shaking speed from 250 to 100 rpm when recombinant *E. coli* cells grew to an OD_600_ of 1.0 at 30 °C at 250 rpm. For bioreactor fermentation, fed-batch culturing was carried out with an initial culture volume of 1.0 L of modified R [27] medium containing 20 g/L glucose, 5 g/L yeast extract, and the required antibiotics in a 3.0-L jar bioreactor BIOSTAT B (Sartorius, Germany) [28]. The temperature was maintained at 30 °C and pH was automatically controlled at 7.0 by adding 5 N NH_4_OH. The DO level was controlled by supplying air or a mixture of air and pure oxygen gas. In order to induce *nar* promoters, cells were grown at DO level > 80% (aerobic phase) until an OD_600_ of 10.0 and then immediately DO level was decreased to < 1–2% (microaerobic). The feeding solution consisting of 800 g/L glucose, 50 g/L yeast extract, 15 g/L tryptone, 15 g/L MgSO_4_·7H_2_O, and 5 g/L KH_2_PO_4_ [29] was periodically added when the residual glucose concentration was below 5–10 g/L. Cell growth was monitored at a wavelength of 600 nm with a SPECTRAmax PLUS384 (Molecular Devices, USA).

### Transcriptional analysis

Cells were grown in LB medium containing 2% (w/v) glucose until mid-exponential growth phase, and total RNA was extracted using Hybrid-R RNA purification kit (GeneAll biotechnology, Korea) according to the manufacturer’s instructions. Quantitative reverse transcription PCR (qRT-PCR) was performed using a Rotor-Gene Q (Qiagen, Germany) and SensiFAST™ SYBR No-ROX One-Step Kit (Bioline, USA). Solutions of 5.0 μL of 2× SensiFAST™ SYBR No-ROX One-Step mix, 0.1 μL of reverse transcriptase, 0.2 μL of RNase inhibitor, 0.4 mM forward and reverse primers (gfpm-qPCR-F and gfpm-qPCR-R), 2.0 μL of isolated total RNA (10 ng/μL), and 1.9 μL of diethylpyrocarbonate (DEPC)-treated water were mixed for each qRT-PCR reaction and qRT-PCR was performed as follow: 45 °C for 10 min, 95 °C for 2 min, and then 40 cycles of 95 °C for 5 s, 60 °C for 10 s, and 72 °C for 5 s. The value of ΔΔ*C*_t_ was averaged from triplicate measurements. The *cysG* gene encoding siroheme synthase was used as a reference gene and the genes expressed by the wild-type *nar* promoter were used as calibrators.

### Western blotting analysis

GFPm expression under the control of *nar* promoters was analyzed by western blotting. The harvest cells were washed and resuspended in 20 mM Tris–HCl (pH 8.0), and then disrupted by sonication. After centrifugation, supernatants were collected, quantified using the Bradford method, and then analyzed by 15% (w/v) SDS-PAGE. For immunodetection of His-tagged GFPm, a monoclonal anti-polyhistidine antibody (Sigma-Aldrich, USA) and horseradish peroxidase-conjugated anti-mouse IgG (Pierce, USA) were used according to the manufacturer’s instructions. GAPDH was used as a reference gene for quantification of proteins.

### Fluorescence analysis

After cells were grown under aerobic conditions, GFPm protein expression under the control of *nar* promoters was induced by lowering DO levels through changing culture rpm of 250–100. Harvested cells were washed and resuspended in 1 mL phosphate buffered saline (PBS, 137 mM NaCl, 2.7 mM KCl, 10 mM Na_2_HPO_4_, and 2 mM KH_2_PO_4_, pH 7.4) and the fluorescence intensity of the reporter GFPm protein was measured using a SPECTRAmax Gemini plate reader (Molecular Devices, USA) with excitation at 470 nm and emission at 510 nm. Cytometric analysis was performed using a BD FACS Calibur flow cytometer (BD Biosciences, USA). GFPm was excited using a 15-mW argon ion laser (488 nm) and fluorescence emission was detected using the FL1 channel (530/30 bandpass filter).

### Metabolite analysis

The concentrations of glucose, d-lactate, 2,3-BDO, and other metabolites were determined using an Agilent Technologies 1200 high-performance liquid chromatography equipped with a refractive index detector (Agilent, USA) and an Aminex HPX-87H column (Bio-Rad, USA) at a flow rate of 0.7 mL/min and column temperature of 50 °C using 4 mM H_2_SO_4_ as the mobile phase.

## Abbreviations

GFP: green fluorescent protein
2,3-BDO: 2,3-butanediol
FACS: fluorescent activated cell sorter
RFU: relative fluorescence unit
